# Twenty-Five Years of AI in Neurology: The Journey of Predictive Medicine and Biological Breakthroughs

**DOI:** 10.2196/59556

**Published:** 2024-11-08

**Authors:** Barak Gutman, Amit-Haim Shmilovitch, Dvir Aran, Shahar Shelly

**Affiliations:** 1 Faculty of Biology Technion - Israel Institute of Technology Haifa Israel; 2 Department of Neurology Rambam Medical Center Haifa Israel; 3 Neuroimmunology Laboratory Ruth and Bruce Rapaport Faculty of Medicine Technion – Israel Institute of Technology Haifa Israel; 4 The Taub Faculty of Computer Science Technion - Israel Institute of Technology Haifa Israel; 5 Department of Neurology Mayo Clinic Rochester, MN United States

**Keywords:** neurology, artificial intelligence, telemedicine, clinical advancements, mobile phone

## Abstract

Neurological disorders are the leading cause of physical and cognitive disability across the globe, currently affecting up to 15% of the world population, with the burden of chronic neurodegenerative diseases having doubled over the last 2 decades. Two decades ago, neurologists relying solely on clinical signs and basic imaging faced challenges in diagnosis and treatment. Today, the integration of artificial intelligence (AI) and bioinformatic methods is changing this landscape. This paper explores this transformative journey, emphasizing the critical role of AI in neurology, aiming to integrate a multitude of methods and thereby enhance the field of neurology. Over the past 25 years, integrating biomedical data science into medicine, particularly neurology, has fundamentally transformed how we understand, diagnose, and treat neurological diseases. Advances in genomics sequencing, the introduction of new imaging methods, the discovery of novel molecular biomarkers for nervous system function, a comprehensive understanding of immunology and neuroimmunology shaping disease subtypes, and the advent of advanced electrophysiological recording methods, alongside the digitalization of medical records and the rise of AI, all led to an unparalleled surge in data within neurology. In addition, telemedicine and web-based interactive health platforms, accelerated by the COVID-19 pandemic, have become integral to neurology practice. The real-world impact of these advancements is evident, with AI-driven analysis of imaging and genetic data leading to earlier and more accurate diagnoses of conditions such as multiple sclerosis, Parkinson disease, amyotrophic lateral sclerosis, Alzheimer disease, and more. Neuroinformatics is the key component connecting all these advances. By harnessing the power of IT and computational methods to efficiently organize, analyze, and interpret vast datasets, we can extract meaningful insights from complex neurological data, contributing to a deeper understanding of the intricate workings of the brain. In this paper, we describe the large-scale datasets that have emerged in neurology over the last 25 years and showcase the major advancements made by integrating these datasets with advanced neuroinformatic approaches for the diagnosis and treatment of neurological disorders. We further discuss challenges in integrating AI into neurology, including ethical considerations in data use, the need for further personalization of treatment, and embracing new emerging technologies like quantum computing. These developments are shaping a future where neurological care is more precise, accessible, and tailored to individual patient needs. We believe further advancements in AI will bridge traditional medical disciplines and cutting-edge technology, navigating the complexities of neurological data and steering medicine toward a future of more precise, accessible, and patient-centric health care.

## Introduction

Neurological disorders are a leading cause of disability and mortality worldwide, affecting millions of individuals and placing a significant burden on health care systems. In 2019, these disorders were responsible for nearly 10 million deaths and 349 million disability-adjusted life-years globally, with stroke and neonatal encephalopathy being the primary contributors [1,2]. Over the past 3 decades, the prevalence of neurological disorders has increased substantially, particularly in low- and middle-income countries, and this trend is expected to continue as populations age [1]. However, we have also witnessed remarkable advancements in technology and data science that are transforming the field of neurology. These developments offer new hope for improving the diagnosis, treatment, and management of neurological disorders. This paper explores the evolving landscape of neurology, focusing on how the integration of cutting-edge technologies and vast datasets is revolutionizing our understanding of neurological disorders and paving the way for more personalized, effective, and accessible care.

In the past 25 years, numerous technological advancements have significantly impacted the field of neurological medicine. These advancements include the integration of cutting-edge imaging technologies that offer deeper insights into brain anatomy, physiology, and function; the use of advanced electrophysiological techniques to create detailed brain region and connectivity maps; breakthroughs in neurogenetics and molecular biology that aid in identifying and characterizing neurological conditions; and the expansion of telemedicine, which allows physicians to deliver more efficient and accessible care.

Specifically, one of the most notable advancements has been the widespread adoption of electronic health records (EHRs). EHRs have not only transformed clinical practice but also opened up vast opportunities for research by creating large datasets that can be analyzed using advanced data science techniques. The integration of EHRs with other data sources, such as imaging and genetic data, has enabled researchers to identify novel disease subtypes, predict patient outcomes, and develop personalized treatment strategies. Another area where technology has made significant strides is in the development of novel diagnostic tools and biomarkers. For example, advances in neuroimaging techniques, such as functional magnetic resonance imaging (fMRI) and positron emission tomography (PET) scans, have facilitated more accurate diagnosis of neurological diseases. Similarly, the discovery of new genomic and molecular biomarkers has paved the way for more targeted therapies and precision medicine approaches. Furthermore, the increasing availability of large-scale neurological datasets, coupled with advancements in machine learning and artificial intelligence (AI), has opened new possibilities for predictive and decision support systems. These tools can assist clinicians in making more accurate diagnoses, predicting disease progression, and optimizing treatment plans based on individual patient characteristics.

**Figure 1:**
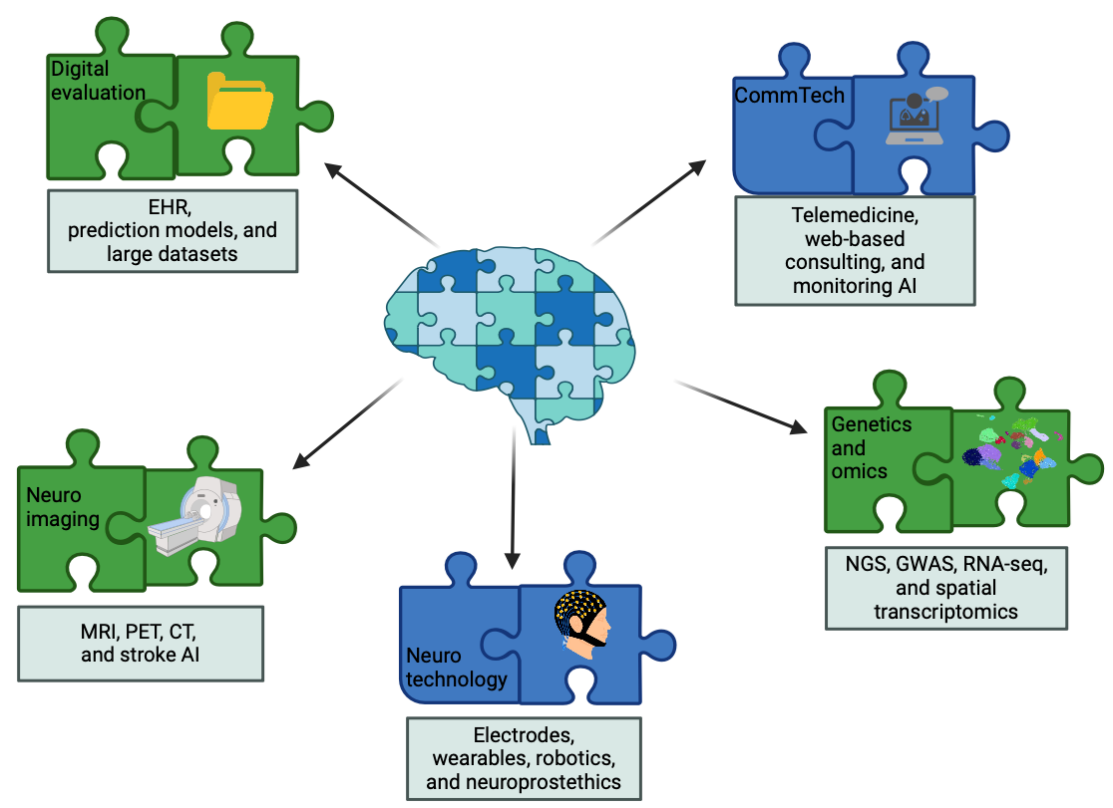
Fields of advances in the last half-century in neurology. AI: artificial intelligence; CT: computed tomography; EHR: electronic health record; GWAS: genome-wide association study; MRI: magnetic resonance imaging; NGS: next-generation sequencing; PET: positron emission tomography; RNA-seq: RNA sequencing.

**Figure 2:**
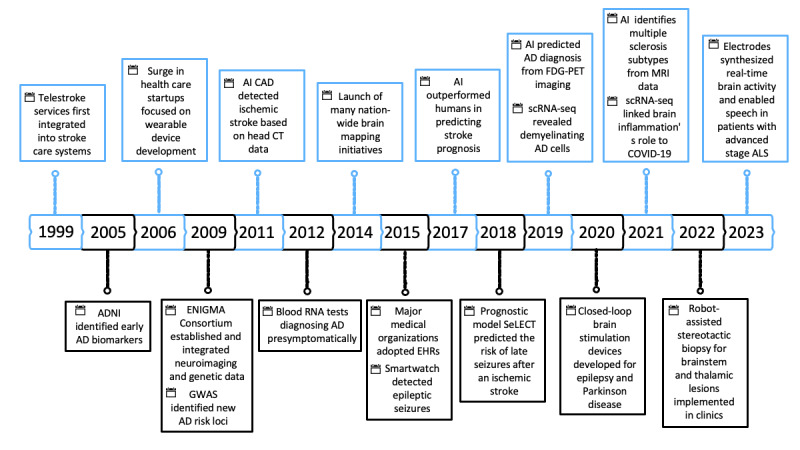
Timeline of key technological advances in the last 25 years. AD: Alzheimer disease; ADNI: Alzheimer’s Disease Neuroimaging Initiative; AI: artificial intelligence; ALS: amyotrophic lateral sclerosis; CAD: computer-aided design; CT: computed tomography; EHR: electronic health record; ENIGMA: Enhancing NeuroImaging Genetics through Meta‐Analysis; FDG-PET: fluorodeoxyglucose-positron emission tomography; GWAS: genome-wide association study; MRI: magnetic resonance imaging; NIH: National Institutes of Health; scRNA-seq: single-cell RNA sequencing; SeLECT: severity of the stroke, large artery atherosclerosis, early seizure, cortical involvement, and territory of the middle cerebral artery.

It is important to acknowledge that while this paper aims to provide a comprehensive overview of the impact of AI on neurology, its scope is necessarily limited. We have focused on key areas that, in our assessment, have most significantly influenced the field of neurology over the past quarter-century. The subsequent 5 chapters of this paper dive deeper into these advancements, exploring how they are reshaping the landscape of neurological care and research (Figures 1 and 2). Rather than attempting an exhaustive analysis of each topic, our goal is to highlight the current state of the art, identify pressing challenges and promising opportunities, and suggest potential future directions within each domain. By doing so, we hope to provide a balanced perspective on the transformative potential of AI in neurology, while also recognizing the vast and rapidly evolving nature of this field. This paper serves as a starting point for further exploration and discussion, acknowledging that the integration of AI in neurology is an ongoing journey with many exciting developments yet to come.

## The Digital Transformation of Neurological Evaluation: From Bedside Physical Examination to Data-Driven Diagnostics

### Overview

The transition from traditional physical examination to digital data acquisition and patient triage in outpatient clinics marks a significant paradigm shift in neurological evaluation. It is widely accepted that a meticulous patient history is crucial for achieving an accurate and timely diagnosis, with estimates suggesting that 70% to 90% of medical diagnoses can be determined by history alone [3]. This, combined with a physical examination and a comprehensive understanding of neuroanatomy, constitutes a traditional approach to neurological diagnosis, primarily aimed at pinpointing the disease’s anatomical location. The specific features that make neurology unique include a heavy reliance on complex physical examination for diagnosis and follow-up, use of specialty-specific neurophysiologic testing (eg, electromyography or nerve conduction studies, electroencephalogram [EEG], sensory evoked potential studies, and sensory evoked potentials), high use of neuroradiologic imaging such as magnetic resonance imaging (MRI) and computed tomography (CT), use of videotaped examinations by clinicians for movement disorders, use of patient-recorded videos or pictures in the medical record (eg, seizures, pseudoseizures, tics, and dyskinesias), and importance of patient documentation of episodic complaints (eg, migraines and seizures).

While traditional approaches have been the backbone of neurological practice, the rapid growth of digital technologies and the increasing volume of patient data have necessitated a shift in how neurologists approach diagnosis and treatment. The digitization of medical records, in particular, has been a game changer, allowing clinicians to capture, store, and analyze vast amounts of patient information in ways that were previously unimaginable. This transition has not only improved the efficiency and accuracy of neurological care but also opened up new avenues for research and discovery.

The adoption of EHRs has been a gradual process, driven by advances in computing technology and the recognition of their potential to improve patient care. The journey began in the 1960s with the earliest attempts to digitize patient information, but it was not until the 1990s that electronic medical records began to gain widespread traction. In the 1990s, the rise of more affordable, powerful, and compact computing technologies, alongside the increasing use of local area networks and the internet, catalyzed the development and adoption of electronic health and medical records, also known as EHRs [4]. Initially, EHRs were predominantly deployed in academic medical facilities, containing only partial medical information, with the remainder still documented on paper [5]. These early systems were mainly hosted on large mainframes with limited functionalities, focusing on laboratory and medication [6]. Their adoption faced challenges due to high costs, data-entry errors, and only partial acceptance by physicians [7]. At this stage, EHRs were primarily used for data interchange among physicians [8] and for image scanning and documentation [9], with clinical use increasing as computers became more integrated into health care as “physician workstations” [10].

There has been rapid adoption of EHRs over the last few years, spurred largely by financial incentives allocated by the Health Information Technology for Economic and Clinical Health Act as part of the American Recovery and Reinvestment Act of 2009. In the years that have since passed, the global use and reliance on EHRs in departments such as the emergency department have grown stronger. By 2015, EHRs had gained recognition from major medical organizations and governments as essential for storing patient data to optimize care delivery [11]. This app endowed the hospital with improved web or client-server–based systems with relational databases, facilitating easier data access and the sharing of medical information through health information exchange networks [12]. This period also saw efforts to standardize EHRs internationally, allowing for a common set of data exchange standards and terminology. In outpatient clinics, there is a consensus that the integration of EHR has resulted in a significant reduction in overall waiting times and a decrease in documentation errors [13].

### Improved Patient Care and Triage in Neurology

**Figure 3:**
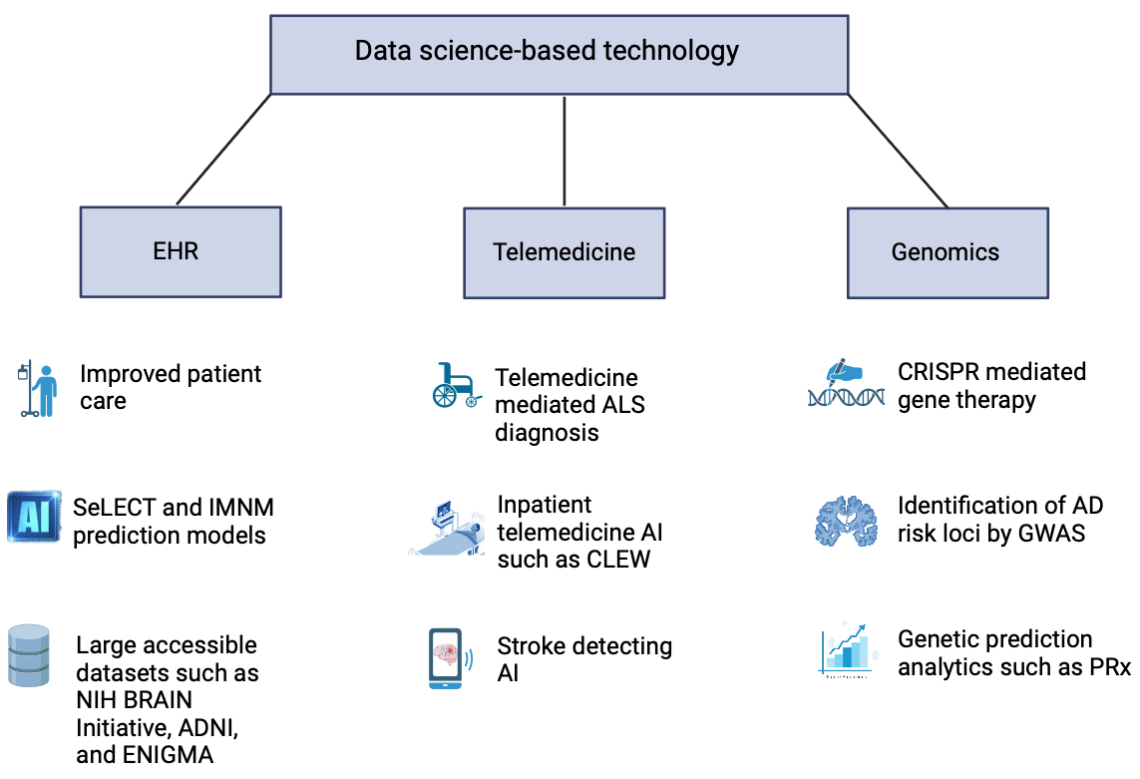
Summary of neurology-related data science–based advances in the last half-century. AD: Alzheimer disease; ADNI: Alzheimer’s Disease Neuroimaging Initiative; AI: artificial intelligence; ALS: amyotrophic lateral sclerosis; CRISPR: clustered regularly interspaced short palindromic repeats; EHR: electronic health record; ENIGMA: Enhancing NeuroImaging Genetics through Meta‐Analysis; GWAS: genome-wide association study; IMNM: immune-mediated necrotizing myopathy; NIH: National Institutes of Health; PRx: pressure reactivity index; SeLECT: severity of the stroke, large artery atherosclerosis, early seizure, cortical involvement, and territory of the middle cerebral artery.

The implementation of EHR system has fundamentally altered both research paradigms and clinical workflows for neurologists (Figure 3). The EHR system, by its very design, has transformed the way neurologists compose clinical notes, often replacing individualized communication styles with template-based entries that aggregate vast amounts of data with minimal effort. Neurologists have written about the challenges of EHR use with many published articles discussing the difficulties in neurology practice. Recent publications report concerns with the efficiency of the use of EHRs in academic practice, challenges of implementation, improper documentation, issues of privacy, and impairing the physician-patient relationship.

The adoption of EHRs has had a significant impact on the field of neurology, along with the broader medical community, influencing everything from clinical practice and patient care to research and administration. EHR systems have standardized the documentation process, making it easier to maintain consistency across patient records. This is particularly beneficial in neurology, where the complexity of neurological conditions requires detailed recording of clinical findings, treatment plans, and patient responses. However, the standardization can sometimes lead to a loss of individual clinician’s nuances in documenting their observations and thought processes, potentially impacting the richness of the clinical narrative. Another area is accessibility and coordination of care, which allows for easier access to patient records across different health care settings, which is crucial for neurology patients who often require multidisciplinary care. This accessibility improves coordination among health care providers, leading to more integrated care plans and better patient outcomes. Furthermore, EHR systems have facilitated the growth of telemedicine, which has become especially important for neurology patients with mobility issues or those living in remote areas. One overwhelming success is the wealth of data captured in EHRs that can be a gold mine for neuroinformatic research and the development of predictive models for neurological diseases. This aspect is particularly relevant in academic and research settings where EHR data can be analyzed to uncover patterns, predict outcomes, and guide the development of new treatment protocols [14].

### Prediction Models Using Electronic Records

In this current era of big data, the focus has shifted toward leveraging the vast databases of EHRs through AI and machine learning technologies. This involves developing AI algorithms for predicting patient risk and personalized treatment plans [15]. One example is a study that focuses on addressing the gap in predicting poststroke seizures, a significant concern in neurology given stroke’s role as a leading cause of acquired epilepsy in adults [16]. The researchers aimed to develop and validate a prognostic model, named the SeLECT score (“Se” severity of the stroke, “L” large artery atherosclerosis, “E” early seizure, “C” cortical involvement, and “T” territory of the middle cerebral artery), for predicting the risk of late seizures (occurring more than 7 days after) in individuals who have had an ischemic stroke. The SeLECT score was developed through a multivariable prediction model using data from 1200 participants in Switzerland and validated externally in 1169 participants across Austria, Germany, and Italy. It incorporates 5 clinical predictors: severity of stroke, large-artery atherosclerotic etiology, early seizures, cortical involvement, and territory of middle cerebral artery involvement. The model’s effectiveness was demonstrated by its ability to stratify the risk of late seizures after stroke with a concordance statistic of 0.77 in validation cohorts, indicating good predictive accuracy. This approach exemplifies the potential of predictive models to transform patient care in neurology by enabling tailored interventions based on individual risk assessments [16].

Another example is a study that introduces a statistical model designed to improve the diagnosis of immune-mediated necrotizing myopathy (IMNM), a condition where delayed diagnosis can lead to significant morbidity. In a subset of IMNM diagnosis is particularly challenging as in patients describe chronic course and lack specific symptoms. The model leverages electrical myotonia versus fibrillations as biomarkers to predict immunotherapy treatment response, based on data from 119 cases of IMNM and 938 other patients with myopathy. All patients underwent electrophysiological evaluations, muscle biopsies, neurological examinations, and creatine kinase measurements [17]. In the broader context of predictive models in neurology, this study exemplifies how statistical models can significantly enhance the diagnosis and treatment of neurological conditions. By identifying specific biomarkers and incorporating them into a predictive framework, such models offer a path toward more personalized and timely interventions. This approach mirrors the potential seen in the SeLECT score for predicting poststroke seizures, further illustrating the critical role of predictive models in advancing neurology practice.

Predictive models may also incorporate other types of data, including imaging, biomarkers, and environmental and lifestyle factors. The scope of predictive models is broad; in cerebral hemodynamics, they focus on assessing cerebral autoregulation to determine the optimal cerebral perfusion pressure for individual patients. For example, the pressure reactivity index [18] uses data from ventricular catheters [19] or intraparenchymal devices [20]. Cerebral metabolism is another area benefiting from predictive analytics, with algorithms analyzing brain interstitial fluid via intracerebral microdialysis to detect metabolic distress, anaerobic metabolism, cell injury, and membrane breakdown. This monitoring facilitates early detection of metabolic changes and guides therapeutic interventions [21]. In addition, predictive analytics plays a critical role in brain oxygenation monitoring, ensuring a balance between oxygen supply and demand. The primary methods in this field include direct brain tissue oxygen tension monitoring [22], jugular venous bulb oximetry [23,24], and near-infrared spectroscopy [25]. Moreover, in recent years, the development of predictive analytics for neurological disorders has seen significant advancements. For instance, researchers have derived a single “Alzheimer Disease Identification Number” from clinical and neuroimaging data, offering a novel approach to tracking disease severity [26]. In multiple sclerosis (MS), a developed predictive model can identify MS subtypes through MRI data and unsupervised machine learning [27]. In Parkinson disease (PD), predictive models have identified antitumor necrosis factor therapy as a potential therapeutic option for mitigating disease risk among patients with inflammatory bowel disease [28]. These advanced analytics methods demonstrate improved accuracy and prognostication over traditional models, offering new insights into patient management and treatment outcomes in neurovascular research.

This shift toward health care institutions taking on the responsibility for developing decision support tools marks a significant point in the regulatory environment and the need for tailored solutions. At the same time, the global health care sector is increasingly tapping into EHR data for AI-based projects, aiming to use the vast amount of medical data to enhance patient outcomes through disease prediction, treatment personalization, and the acceleration of new drug discovery especially in neurology. These efforts require strict protocols for data standardization, processing, and privacy to maximize the benefits of AI research while protecting sensitive patient information. Supported by initiatives such as those from the Korean government [29,30], there is a growing movement toward leveraging AI in health care, pointing toward a future where AI, powered by EHR data, becomes central to advancing medical research and delivering personalized care to patients.

### Creation of Large Accessible Datasets

Despite the critical importance of training databases, there is a lack of publicly accessible, reliable datasets. This shortage primarily results from data sharing barriers across institutions, the time and cost of data annotation, and occasionally, the complexity of building data processing pipelines. Training data may be preannotated, a process known as “supervised learning,” or it may not be, which is referred to as “unsupervised learning.” In the realm of AI in health care, supervised learning models are predominantly used due to the critical nature of their applications, where human lives hinge on the accuracy of AI outputs. To address this issue, several national and multinational data banks have emerged, covering various neurological conditions [31]. From 2013 to 2014, several governments initiated national initiatives aimed at understanding brain function, such as the National Institutes of Health (NIH) BRAIN Initiative [32], the Human Brain Project [33], and the Brain Mapping by Integrated Neurotechnologies for Disease Studies project in Japan [34]. Many of them soon became global and involved collecting and analyzing voluminous data, including neuroimaging, genetic, biospecimen, and clinical assessments, to unlock and decipher the genesis and prognosis of neurological conditions. As the collection of data became increasingly prominent, the need for procedures, standards, hardware, and software for data-intensive computing increased [35]. These projects leverage big data to explore the brain structure (“connectome”) and function with the ultimate goal of developing new treatments for neurological diseases.

For instance, the Alzheimer’s Disease Neuroimaging Initiative (ADNI), which was launched in 2005, aims to identify biomarkers for the early detection and monitoring of Alzheimer disease (AD). It supports interventions, prevention, and treatments through early diagnostics and facilitates global data sharing [36,37]. By collecting and analyzing data on cognitive functions, brain structure, metabolism (via PET and MRI scans), genetics, and biochemical changes in a diverse cohort, ADNI has provided significant contributions. Its most substantial contribution to date is the development of methodologies for the early diagnosis of AD using biomarker tests, such as amyloid PET scans and cerebrospinal fluid lumbar punctures. This approach has revealed a significant number of individuals in their mid-70s showing preclinical stages of AD [38], underscoring the critical importance of early prevention and treatment strategies for the disease.

In parallel, the Enhancing NeuroImaging Genetics through Meta‐Analysis Consortium [39], established in 2009, represents another pivotal big data initiative in the field of neuroscience. It aims to integrate neuroimaging and genetic data to investigate brain genotype-phenotype relationships. Notable achievements of the Enhancing NeuroImaging Genetics through Meta‐Analysis consortium include identifying genome-wide variants related to brain imaging phenotypes [40,41] and examining MRI-based abnormalities across various conditions [39,42] such as major depressive disorder [43] and bipolar disorder [44]. These discoveries have substantially improved diagnostics and patient care, showcasing the value of integrating big data in advancing neuroscience research.

Beyond the contributions of major organizations, recent years have witnessed the emergence of numerous big data–driven diagnostic solutions in neuroscience from smaller entities. Key discoveries include the identification of MS subtypes using sophisticated imaging analyses and improvements in MRI data [27] and unsupervised machine learning as well as the differentiation of dementia subtypes through the analysis of multimodal data from ADNI [45]. In addition, significant strides have been made in depression research, highlighted by the successful prediction of treatment response through various methods: connectome gradient dysfunction paired with gene expression [46], resting state connectivity markers of transcranial magnetic stimulation response [47], and a sertraline-response EEG signature [48]. In terms of migraine research, the Italian Migraine Registry is being developed to serve as a comprehensive source of clinical, biological, and epidemiological big data. This registry aims to enhance our understanding of therapeutic response rates and the efficacy of treatments [49]. Another notable diagnostic initiative is the iPrognosis mobile app designed to expedite the diagnosis of PD and improve the quality of life for patients with PD. The app functions by collecting data during the user’s interaction with smart devices, including smartphones and smartwatches, showcasing the innovative use of technology in patient care and research [50].

In summary, over the past decade, numerous emerging AI technologies have significantly enhanced patient flow through various means. These advancements range from facilitating direct intrahospital communication to autonomously analyzing radiological images and assisting in the selection of patients for specialized treatments. The development and refinement of these AI systems rely heavily on access to extensive data banks, which serve as foundational resources for training purposes. Such repositories, both large and small, have already yielded substantial improvements in the diagnosis of numerous neurological conditions. The trend toward leveraging big data is expected to intensify, with the emergence of larger databases in the coming years. This expansion will be further supported by an increasing volume of data collected through wearable technology. Consequently, these databases will play a crucial role in enabling the development of new AI-driven approaches for treatment and diagnosis [51].

## New Communication Technologies: Telemedicine and Remote Patient Monitoring

### Overview

As powerful an approach as AI-mediated medical treatment is, it still does not fully address the growing demand for neurological care, which is exacerbated by a shortage of neurologists. This challenge is expected to intensify with the expanding aging population, highlighting the urgent need for a more substantial neurological care provision. Telemedicine emerges as a promising solution to bridge this gap, offering access to those hindered by geographical or physical barriers such as mobility issues (Figure 3). It facilitates earlier access to specialized care, potentially reducing the strain on patients and caregivers while enhancing patient satisfaction. In addition, telemedicine provides an avenue for neurologists facing social, physical, or health-related challenges to maintain or extend their practice, including those considering part-time work or retirement. It also allows for more efficient use of neurologists’ time by eliminating travel between facilities, thereby increasing their availability for patient evaluations and the ability to serve remote clinics. Telemedicine leverages a wide array of technologies, including 2-way videoconferencing, data storage and forwarding, and mobile and wireless devices, to deliver care more flexibly and efficiently [52]. Despite previous barriers to telemedicine adoption, such as reimbursement issues, recent policy changes by the Centers for Medicare and Medicaid Services, which expanded Medicare-covered telehealth services for 2019, have significantly improved access to neurological care. These changes not only enhance patient care options but also open new revenue streams for neurologists, signaling a shift toward a more accessible and sustainable model of neurological care delivery [53,54].

### Virtual Consultations

Telestroke services, first described in 1999 and formally integrated into stroke care systems for over a decade, have significantly influenced the broader field of telemedicine. This period has seen expanded access to care, improvements in quality, and higher rates of reperfusion therapy for patients with ischemic stroke [55]. In addition, comparisons between telestroke and in-person evaluations have shown similar rates of stroke mimics, indicating that assessment scales and imaging interpretations are just as effective when conducted remotely [56]. Telestroke’s acceptance across diverse cultures further underscores its effectiveness and potential for broader application. However, despite these strides in enhancing stroke care through telemedicine, there remains a gap in data regarding the suitability and practicality of telemedicine for treating other neurological conditions [57], highlighting an area ripe for exploration and development.

Another example is in neuromuscular conditions that encompass a wide range of disorders, from common diabetic neuropathy to rare diseases such as periodic paralysis. Many of these conditions, including amyotrophic lateral sclerosis (ALS), necessitate a comprehensive, multidisciplinary management approach. Despite rapid advancements in diagnostic technologies, the accurate diagnosis of many neuromuscular disorders often hinges on detailed neurological examinations to detect subtle clinical signs that might be overlooked in teleneurology assessments. However, telemedicine has been found beneficial for patients with established diagnoses and stable symptoms, offering a convenient option for follow-up evaluations [58]. Research on the use of teleneurology specifically for neuromuscular disorders is limited, and only a handful of studies have been published to date. These studies, primarily focused on patients with a confirmed diagnosis of ALS [55], revealed a generally positive perception of teleneurology among patients, caregivers, and health care providers. Patients expressed high levels of satisfaction appreciating particularly the elimination of travel-related burdens, which led to less stress and more comfortable medical interactions. In addition, a smaller study involving patients with advanced facioscapulohumeral muscular dystrophy indicated that teleneurology was well-received by both patients and caregivers [57]. The quality of care provided via teleneurology was rated highly in patient questionnaires, although it was noted that acute care issues were not addressed in these evaluations.

There are other examples such as concussions and traumatic brain injuries [59,60], dementia evaluations [61], and the management and follow-up of patients with epilepsy [62,63]. It has also facilitated the diagnosis and treatment of nonacute headaches [64-66], assessment of movement disorders [67-69], remote care for MS [70,71], as well as follow-up consultations and care management patients with neuromuscular diseases [52,72]. The COVID-19 pandemic saw an increase in telemedicine use across specialties to manage patient care during lockdowns, mask mandates, and overall minimization of personal interactions. This era is characterized by the adoption of telemedicine on a national scale, exemplified by Saudi Arabia's adoption of telemedicine as an alternative health delivery system [73] and the launch and deployment of a telemedicine program by the Italian government [74].

### Improving Patient Flow and Early Detection

Stroke is a highly prevalent neurological disorder, affecting approximately 9.4 million Americans aged >20 years between 2017 and 2020 [75]. More than half of the patients who experienced stroke were left chronically disabled [76], and the annual mortality rate as of 2020 was 160,000 Americans [75]. For many years, stroke diagnosis was significantly hampered by time delays between the initial detection at radiologic centers and subsequent treatment at thrombectomy centers within hospitals. As of 2016, this delay averaged nearly 100 minutes in the United States, leading to increased morbidity [77] and disability [78]. In response to this challenge, an AI company developed a convolutional neural network (CNN) algorithm capable of automatically detecting ischemic stroke patterns associated with large vessel occlusions (LVOs) [79]. Upon identifying an LVO, the algorithm autonomously alerts the stroke treatment team, bypassing the need for any intervention by the clinician who requested the radiologic examination. Alerts are dispatched through a mobile app, facilitating immediate communication and resulting in an average reduction of 52 minutes in the time to LVO treatment initiation [80]. Beyond facilitating direct communication, modern AI-based telestroke systems enhance patient flow by autonomously analyzing radiological images, often surpassing the capabilities of human radiologists [81]. Clinical AI today is adept at interpreting CT and MRI scans to determine the size and extent of brain damage caused by ischemic strokes [81] and can even forecast the potential progression of the stroke [82].

AI has also augmented radiologist performance by aiding in the selection of patients for endovascular thrombectomy. This is achieved through the integration of automated Alberta Stroke Program Early CT Scores (ASPECTS) with clinical presentations, thereby correlating with the NIH Stroke Scale scores [83,84]. Moreover, AI proves its proficiency in acute prognosis prediction by evaluating detected infarct volumes [85] or white matter hyperintensities [86]. It even enables predicting treatment outcomes [84,87,88] with remarkable accuracy, including a notable 7% improvement in forecasting symptomatic intracerebral hemorrhage [89]. These advancements highlight AI’s broad application in stroke care, from the analysis of radiological imaging to the identification of stroke indicators, enhancing intrahospital communication and significantly contributing to the decision-making process for timely and effective treatments.

### Quantum Computing

Quantum computing represents the potential to change the way we view data storage, specifically in neurology. Unlike classical computers, which use bits to process information as binary 0s or 1s, quantum computers use qubits that can exist in multiple states simultaneously due to superposition. This allows quantum computers to process vast amounts of data at unprecedented speeds, making them exceptionally powerful for complex computations. Quantum computing could significantly enhance our ability to analyze large datasets, such as those generated from neuroimaging, genomics, and EHRs. The ability to process and analyze these massive datasets more efficiently can lead to more accurate models of brain function, disease progression, and treatment outcomes. For example, quantum algorithms could optimize machine learning models used for diagnosing neurological disorders, predicting disease trajectories, and personalizing treatment plans.

In summary, telemedicine and remote patient monitoring have emerged as transformative forces in neurological care, offering unprecedented access, convenience, and efficiency. From virtual consultations for stroke, neuromuscular disorders, and movement disorders to AI-driven early detection and prognostication in acute stroke management, these technologies are reshaping the landscape of neurological services. As we navigate the challenges posed by an aging population and a shortage of neurologists, the continued adoption and advancement of telemedicine hold immense promise in ensuring timely, equitable, and effective care for patients with neurological conditions worldwide.

## Genetics and Omics: Driving Personalized Medicine in Neurology

### Overview

The genetic and molecular insights gained from omics studies are informing the development of neurotechnological interventions, from brain-computer interfaces (BCIs) to neuroprosthetics (Figure 3). Despite the historical limitations imposed by the high costs of genetic analysis and the constrained ability to address neurological disorders once identified, recent technological advancements in DNA sequencing and gene editing have propelled genetic analysis and gene therapy to the forefront of clinical neurology. These innovations promise significant improvements in patient care, emphasizing the critical role of genetics in understanding and managing neurological diseases. The human genome’s complexity, with its 3 billion nucleotides, of which <2% encode proteins, underlines the intricate relationship between genetic variations in both protein-coding genes and noncoding regulatory DNA and disease risk. Diseases can be monogenic, resulting from a single gene mutation, or polygenic, involving mutations across multiple genes. The advent of next-generation sequencing (NGS) has exponentially increased the speed, accuracy, and affordability of DNA sequencing, making it possible to use an individual’s genome to guide their medical care. This leap in sequencing technology, alongside developments in gene editing, particularly Clustered Regularly Interspaced Short Palindromic Repeats (CRISPR)/Cas9, marks significant advancement toward correcting genetic mutations responsible for neurological diseases.

The integration of genomic information from genome-wide association studies (GWASs) for predicting disease risk and aiding in the identification of patient populations for clinical trials, points toward a future where genetic screening plays a crucial role in early intervention strategies. As gene-based diagnostics and treatments become more accessible and refined, the potential for addressing a vast array of neurological conditions grows, underscoring the importance of continued investment in basic science research to fuel the development of tomorrow’s treatments.

### Genetics in Predictive Analytics

Together, NGS and GWAS significantly enhance neurological predictive analytics by offering a comprehensive approach to understanding complex diseases. NGS provides detailed genetic screening and diagnosis, enabling precise prognostication, informed treatment planning, and accurate genetic counseling through genetic risk scores. It deepens our understanding of disease mechanisms by mapping phenotype or genotype correlations, paving the way for novel therapies and personalized medicine in neurology. GWAS, in turn, identifies genetic loci associated with various neurological conditions, illuminating their heritability and pathophysiology and revealing potential therapeutic targets. Collectively, these technologies form a potent toolkit for elucidating the genetic foundations of neurological disorders, promising advancements in treatment and patient care [90-92].

In the past 2 decades, GWAS facilitated many discoveries, such as the identification of multiple novel risk loci in neurodegenerative diseases like AD and PD (CR1, BIN1, and PICALM for AD [93]; SNCA and MAPT for PD [94]) that elucidate roles in lipid processing, the immune system, and synaptic-cell functioning pathways. Similarly, in ALS, GWAS findings have highlighted genes such as UNC13A and the significance of the 9p21.2 region in both familial and sporadic forms [95,96]. Chronic conditions such as MS [97], epilepsy [98], and restless legs syndrome [99] have benefited from GWAS revealing numerous loci, particularly highlighting the autoimmune nature of MS and the dopaminergic neurotransmission and iron dysregulation in restless legs syndrome. Cerebrovascular disorders, such as stroke [100] and Moyamoya disease [101], have revealed specific genetic risk factors through GWAS, including the identification of 8 different loci causing neurological instability postischemic stroke and the strong association of the RNF213 locus with Moyamoya disease risk. These discoveries underscore the complexity of neurological diseases and the crucial role of genetic factors, paving the way for targeted therapies and improved diagnostic strategies.

Genetic insights are instrumental for predictive models that use algorithms and statistical techniques, including machine learning and neural networks, to identify patterns and predict future clinical outcomes from data. For example, in neurovascular conditions such as cerebral aneurysms [102] and arteriovenous malformations [103], predictive models have successfully forecasted risks of cerebral aneurysm rupture and outcomes following endovascular treatment of arteriovenous malformations. These predictions are based on a combination of basic demographics, clinical information, and computational blood flow simulations processed through machine learning and image processing techniques.

Beyond DNA sequencing, other “omics” have been transformative in various neurology traits. For instance, transcriptome analysis, used to measure the expression levels of genes, provided significant insights across various diseases. In AD, it uncovered 3 molecular subtypes [104] and led to the development of a blood RNA test that distinguishes AD from other dementias before symptom onset [105,106], also highlighting the downregulation of NeuroD6 as a potential biomarker [107]. For PD, it enabled patient stratification based on mitochondrial or lysosomal dysfunctions and assisted in selecting neuroprotective compounds [108]. In ALS, transcriptome analysis facilitated the molecular classification into 2 distinct subtypes, sporadic ALS group 1 and group 2, by analyzing deregulated genes and pathways in postmortem cortex transcriptomes [109,110]. Moreover, it revealed central nervous system (CNS) dysregulation of over 300 biological processes in prion infections and suggested alternative pathways for astrocyte activation [111]. It also identified molecular heterogeneity in trigeminal ganglia subregions, aiding in the understanding of migraine and headache mechanisms through the analysis of postmortem trigeminal ganglia [112]. Even though it was revolutionary at the time, bulk transcriptome analysis has several disadvantages that have prompted the development and adoption of single-cell RNA sequencing (scRNA-seq) and spatial transcriptomics technologies. One key limitation of bulk transcriptomics is its inability to capture cellular heterogeneity within complex tissues or cell populations, as it provides only an average expression profile from a bulk sample. This averaging masks the diversity of individual cell types and their unique transcriptional states, which are crucial for understanding biological processes and disease mechanisms. In addition, bulk transcriptomics lacks spatial context, meaning that it cannot pinpoint where specific genes are being expressed in a tissue. scRNA-seq addresses these issues by profiling the transcriptomes of individual cells, revealing the cellular heterogeneity and enabling the identification of rare cell types and subpopulations. This detailed cellular landscape often yields valuable insights into disease progression, such as the discovery of key demyelinating subpopulations mediating early AD progression [113]. These discoveries are made possible and reliable due to large scRNA-seq databases, which can be either self-generated or obtained from publicly available repositories and atlases. The past decade has witnessed the emergence of many such atlases, including the Allen Brain Atlas [114], making it easier for researchers to conduct scRNA-seq analysis, as self-sequencing of data may be time-consuming and costly. Spatial transcriptomics technologies go a step further by retaining the spatial context of gene expression, allowing researchers to map where in a tissue each gene is active. Together, scRNA-seq and spatial transcriptomics offer a more detailed and nuanced view of gene expression.

Selective neuronal vulnerability as a subfield in neurology focuses on the molecular mechanisms underlying enhanced neuronal degeneration. This field highly benefited from single-cell technologies, which linked tau accumulation to AD progression and depletion of specific excitatory neurons [115]. In addition, these technologies enabled the identification of molecular features pruning degradation of dopaminergic neurons in PD [116]. Moreover, it facilitated the characterization of specific hippocampus and dorsolateral prefrontal cortex neuronal subtypes, involved in many neuropsychiatric disorders, such as schizophrenia and major depressive disorder [117]. Another neurology subfield highly benefiting from scRNA-seq-based discoveries is neuroimmune dysfunction, which sheds light on the function of immune cells in neurodegenerative disease progression. For example, studies have identified disease-associated microglia with unique gene expression profiles in AD models and distinct microglial responses associated with different stages of neurodegeneration [118,119]. In MS, transcriptomic analyses have uncovered microglial subtypes with specific gene dysregulations, suggesting potential therapeutic targets [120]. Furthermore, the adaptive immune response, involving T cells and B cells, has been implicated in the pathology of MS and PD, highlighting the influence of immune cells on neuronal degeneration [121,122]. Single-cell sequencing has also revealed key insights into glioma, showing how myeloid cell interactions within the tumor microenvironment drive disease progression and affect treatment outcomes [123]. Similarly, in COVID-19, it has identified changes in microglia and astrocytes gene expression, suggesting that inflammation and immune responses contribute to neurological symptoms, opening new paths for treatment [124]. Finally, single-cell technologies have also revolutionized the understanding of how different cell types within the CNS and tumors respond to treatments. In glioblastoma [125] and medulloblastoma [126], for instance, scRNA-seq has identified potential therapeutic targets based on the cells’ developmental and inflammatory processes, demonstrating the potential for tailored treatments.

Despite significant advancements in the field, single-cell transcriptomics still faces challenges and limitations. These include technical hurdles in sample collection [127] and cell isolation from brain tissue [128,129], which impact data quality and reproducibility. Key future directions involve advancing single-cell multiomics to integrate various data types with clinical information, enhancing the precision of spatial transcriptomics and applying these technologies to brain organoids for deeper insights into brain function and pathology.

## Neurotechnology: Advancing Diagnosis, Treatment, and Rehabilitation in Neurology

### Overview

**Figure 4:**
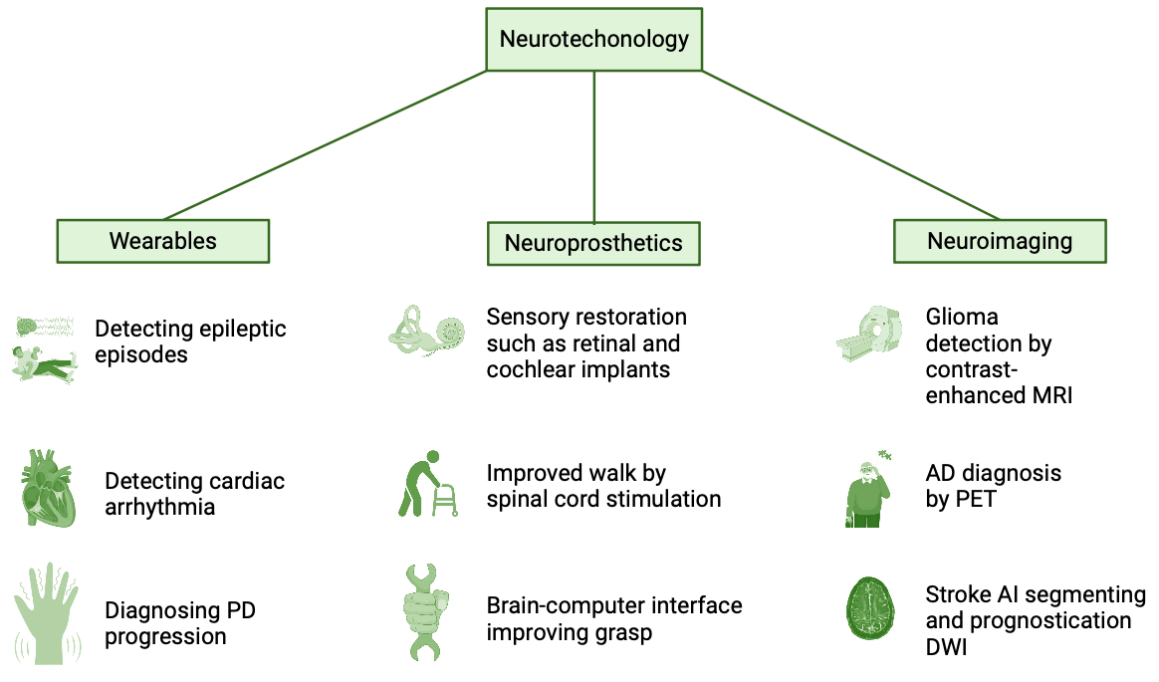
Summary of neurotechnology advances in the last half-century. AD: Alzheimer disease; AI: artificial intelligence; DWI: diffusion-weighted imaging; MRI: magnetic resonance imaging; PD: Parkinson disease; PET: positron emission tomography.

Neurotechnology refers to the integration of techniques and devices facilitating a direct interface between technical apparatuses and the nervous system (Figure 4). These technical components, including electrodes, computers, or advanced prostheses, serve the purpose of capturing signals from the brain and converting them into operational commands or modulating brain activity through the application of electrical or optical stimuli. Ongoing research explores closed-loop interactions between systems for signal acquisition and stimulation (control circuits) [130].

Neurotechnology has its roots in the early exploration of brain electrical activity. Electrical currents of the brain were first described in 1875 by Richard Caton, who observed electroencephalography from exposed rabbits’ and monkeys’ brains. In 1924, German neurologist Hans Berger enhanced the measurement of human brain electrical activity through the scalp, recording and depicting it graphically on paper. This laid the foundation for modern EEG technology, which has become a cornerstone of noninvasive BCIs. EEG signals are now commonly used in BCIs to facilitate bidirectional communication between the human brain and external devices. By monitoring various cortical regions, it is possible to extract signals across multiple frequency bands associated with distinct human behaviors, enabling the study of corresponding patterns. EEG-based BCIs have significantly advanced our understanding of cognitive activities and contributed to progress in computer science and engineering [131]. In EEG-based BCI applications, machine learning technologies typically fall into 2 major categories: classification and individual adaptive tasks. Deep learning, a subset of machine learning, uses deep neural networks to learn EEG patterns [132], featuring numerous neurons across multiple layers to capture cognitive-related features. The potential of BCIs is particularly evident in the field of robotics. EEG-based BCIs have demonstrated efficacy in communication with robots, with early applications, including the control of wheelchairs through visual simulations or motor imagery [131]. These advancements pave the way for more sophisticated neurotechnological interventions in movement, language, and speech, as we will explore in the subsequent sections.

In summary, as neuroprosthetics involves devices that interact directly with the nervous system to restore or enhance neural functions and BCIs enable direct communication between the brain and external devices, they can bypass traditional pathways. They typically rely on electrodes that capture electrical signals from the brain or stimulate neural tissue. These electrodes can be noninvasive, which are placed on the scalp, or invasive, which are implanted directly into the brain. BCIs decode neural signals into commands that control prosthetic limbs, computers, or other devices, often using machine learning algorithms to interpret complex brain activity. There are a few technical challenges such as ensuring long-term biocompatibility and stability to prevent immune responses and device degradation. The integration of neurotechnology into clinical practice requires extensive training for both patients and health care providers. Patients must learn to use and control neuroprosthetic devices effectively, which often involves cognitive and physical training. Health care providers need specialized knowledge to implant, configure, and maintain these devices, as well as to provide ongoing support and adjustments based on patient needs.

### Aiding Movement Language and Speech

One example is electrodes that are capable of noninvasively capturing electrical fields generated by the active brain, typically facilitated by their placement on the surface of the head in the form of electrode caps. This method is deemed noninvasive as the electrodes do not penetrate body tissues. Notably, it finds application in patients afflicted with ALS, particularly during the advanced, near-complete paralysis stages where it aids speech by synthesizing it real time directly from brain activity [133]. For instance, electrodes used for deep brain stimulation (DBS) are meticulously inserted by neurosurgeons into targeted brain regions. Through modulation of these targets, it becomes feasible to suppress or ameliorate certain symptoms associated with various brain disorders. For instance, DBS serves as a therapeutic option for patients with PD when conventional medication proves ineffective. While DBS does not offer a cure or halt the progression of neurodegenerative processes, it substantially alleviates hallmark symptoms such as tremors or rigidity, thereby significantly enhancing patient well-being and overall quality of life [130].

### Neurological Wearables

Another illustration within the realm of neurotechnology pertains to neurological wearables. A primary challenge concerning wearable sensors revolves around creating stretchable and skin-attachable electronic devices capable of seamlessly and inconspicuously monitoring human activity and vital signs without impeding or restricting the user’s movement [134]. The initial breakthrough in implantable medical devices came with the development of a pacemaker for patients with arrhythmia in 1958 [135]. Since this milestone, a range of pacemakers and implantable cerebellar stimulators have been developed and used [134].

In neurology, wearable devices are considered an evolving technology used to track and monitor the patient’s ambulatory status for long periods. It can track vital signs and other types of data, creating a digital profile of the patient [136], even during their sleep. The collected data are expected to improve the diagnosis, assessment, and treatment of patients with various conditions. Out of the health care companies investing in the development of wearable devices, around 60% were founded after 2006, whereas the oldest one was founded in 1993 [137]. Many wearable devices were developed to diagnose, monitor, and treat neurological disorders, such as stroke, PD, and epilepsy. In stroke, smartphones and smartwatches are primarily used to monitor parameters, such as upper extremity activity, walking, and physical activity [138], all of which may detect pulse and cardiac arrhythmia [139] that can cause subsequent transient ischemic attacks or ischemic strokes [140]. In PD, wearable devices in the form of sensors attached to the lower back may track cardinal motor symptoms, such as postural sway, tremor and bradykinesia, quantity and duration of freezing and falling phases, sleeping distributions, and also more cognitively advanced symptoms like dyskinesia [138]. In epilepsy, most wearables are wrist-worn sensors with accelerometers, which are used to identify seizures based on movement patterns that might be associated with tonic-clonic or convulsive seizures [138].

One example of such a device is the Embrace smartwatch, which quantifies alterations in skin electric conductance that correspond to epileptic activity within the brain, and it can notify contacts or caregivers about the seizure activity it identified [141]. Other such devices include one that had electrodes attached to the biceps and detected tonic-clonic seizures and another that detected simple partial seizures [141].

An additional instance pertains to the use of wearables within the realm of sleep neurology. The neurological status of patients is notably influenced by both the quality and quantity of sleep. Wrist-worn actimetry sensors have been established as longstanding tools in sleep studies, enabling the monitoring of various physiological parameters associated with sleep [140]. These devices may be supplemented by cardiac monitoring or mattress-based devices, colloquially termed “nearables,” which possess the capability to track respiratory movements [142]. Several newer devices actively intervene with the patient’s sleep, helping them to get better sleep quality [140].

In all the mentioned wearables, AI technologies play a crucial role in their embedded architecture, as they enable the mass analysis and process of the detected data. However, efficient AI tools or wearables require large amounts of training data, effective noise removal from detected features, and subsequent feature selection (ie, focusing on important data characteristics for each type of evaluation). Furthermore, it involves distinguishing between similar activities and developing computationally efficient algorithms and hardware implementations. Nevertheless, there are significant technical challenges such as the need for energy-efficient designs to ensure long battery life, robust data privacy and security measures, and overcoming the devices’ limitations of real-time data processing. Addressing these challenges is essential for the successful deployment and widespread adoption of AI-enabled neurological wearables [143,144].

Despite its ongoing popularity and contribution to patient care, there are some current issues with wearables in the field of neurology. These mainly include a lack of high-quality data, an absence of accepted evaluation standards, and limited implementation strategies; many wearables lack robust efficacy data that would improve the care of abundant disabling neuropsychiatric conditions, such as migraine and depression [140]. As for evaluation standards, even though the American Psychiatric Association proposed a framework for evaluating digital health tools in 2018 [145], there is still no widely accepted standard [146]. This lack of standards results in inconsistent evaluations and limits implementations. Apart from evaluation problems, the rapid development of wearables also outpaces the creation of validation protocols, resulting in a lag in adapting these tools to health care systems [140].

### Robotics in Neurological Diseases

Researchers have discovered that robotic devices significantly enhance stroke rehabilitation by offering patients tailored, intensive, and repetitive training. These devices facilitate real-time feedback, enabling immediate correction of movement errors, thus fostering more efficient and effective motor learning. With the ability to provide targeted training, these robots not only enhance motor learning but also deliver objective performance and function measurements. Customizable to individual patient needs, these robotic systems can be programmed for specific training or therapeutic interventions, underscoring their versatility in rehabilitation [147]. Neural rehabilitation is an emerging field aiming to restore defective neurological circuits’ functionality or enhance the remaining functionality of impaired ones. Its purpose is to enhance patient’s independence and improve their quality of life by relying on the principle of neuroplasticity [148,149], a subject that relies on the idea that CNSs and peripheral nervous systems can be retrained after an injury to achieve an effective rehabilitation [150]. Another application is in pediatric neurosurgery; robot-assisted stereotactic biopsies for brainstem and thalamic lesions have proven effective, showcasing the potential of robotic procedures in enhancing surgical precision and accuracy and minimizing tissue damage. This approach has been particularly promising in pediatric neurosurgery, offering a method to improve outcomes while reducing the risk to surrounding healthy tissues [147].

Robotics is also making strides in the clinical assessment of neurological disorders and upper-limb therapy, indicating a broader application of this technology beyond traditional settings. Investigations into robot-assisted diagnosis and the use of robotic control through neural interfaces for individuals with tetraplegia highlight the innovative applications of robotics in neurology and intensive care.

Human-robot interaction is an emerging field that integrates AI, robotics, and social sciences to facilitate interaction and communication between humans and robots. It has various applications in medicine and also specifically in neurorehabilitation [151,152]. When using robots, numerous considerations must be considered, including safety, learning by demonstration, imitation learning, cognition and reasoning, perception, and more [153]. Typically, AI algorithms and systems are used to address these issues, thereby enhancing the overall interaction and experience for patients. Owing to the presence of multiple representations within an environment, there is often an abundance of multimodal data, such as visual, audio, infrared, and ultrasound inputs. These inputs are used by AI algorithms capable of conducting tasks, such as object classification, prediction, and task planning [154].

### Neuroprosthetics

Neuroprosthetics is an evolving field that combines neuroscience, engineering, and medicine to develop devices that can restore or enhance neural function. These devices, known as neuroprostheses, interact directly with the nervous tissue, usually to bridge the gap between lost function and the brain’s control over the body. Over the past 25 years, several key advancements have propelled the field of neuroprosthetics forward. Notable examples of these advancements are BCIs, which have emerged as a promising avenue for restoring communication and control in patients with severe motor impairments. Notable achievements include the development of high-performance BCIs that enable users to control prosthetic limbs with near-natural dexterity and speed. One example of such achievement is a description of 2 patients with spinal cord injury who were able to regain their grasp function through neuroprosthesis, by using an asynchronous BCI, allowing them to complete a Grasp and Release Test of the paperweight multiple times [155].

Another example is advancements in sensory restoration. Neuroprosthetics has made significant progress in restoring sensory function, particularly in the realm of hearing and vision. Cochlear implants, which aid more than half a million people worldwide with severe to profound hearing impairment [156], electrically stimulate the auditory nerve to restore hearing and have become increasingly sophisticated, offering improved sound quality and speech recognition. Recently, a demonstration of a new development in the cochlear implant field was presented, allowing better pitch perception for the users by using haptic stimulation on the forearm [156]. Similarly, retinal implants and optogenetic approaches have shown promise in restoring visual perception in individuals with blindness, as can be seen from the launching of 2 electrical retinal prostheses in the last 2 decades, as well as preclinical and early clinical trials of gene therapies in the field of optogenetics [157].

Neuroprosthetic devices have also been developed to stimulate the spinal cord, offering new treatment options for individuals with spinal cord injuries or neurological disorders. These devices can modulate neural activity in the spinal cord, leading to improved motor function, reduced spasticity, and enhanced sensory feedback. Recent studies have demonstrated the potential of spinal cord stimulation to restore voluntary movement in patients with paraplegia. Another demonstration is of functional electrical stimulation electrodes that help patients with improper trunk stabilization due to spinal cord injuries by stimulating lumbar erector spinae among other muscles, improving their posture and forward reach and easing their transfers [158,159]. Furthermore, epidural spinal cord stimulation has been proven to activate central pattern generator for locomotion, thus improving walking in patients with incomplete spinal cord injuries [160].

Neuroprosthetics have led to the development of closed-loop systems that can sense and respond to neural activity in real time. These systems incorporate feedback mechanisms that allow the device to adapt its stimulation parameters based on the user’s needs and intentions. Closed-loop neuroprosthetics have shown promise in applications, such as tremor suppression in PD and seizure detection and intervention in epilepsy. A neurotechnology company created a closed-loop stimulation device that can detect and prevent seizures from 4 channels [161], by comparing preseizure parameters to predefined thresholds, by both cortical and deep-brain stimulation. It also reduced sudden unexpected death in epilepsy significantly [162]. Another company developed a closed-loop device for PD treatment that can record focal deep-brain potentials and accordingly adjust the stimulation amplitude and frequency [163].

Finally, neuroprosthetic devices have become increasingly miniaturized and wireless, improving their implantability and reducing the risk of complications. Wireless power transfer and data communication have enabled the development of fully implantable systems that can operate without the need for external hardware. These advances have greatly enhanced the practicality and acceptability of neuroprosthetic devices for long-term use. One example of miniaturized neuroprosthetics is cochlear implants, which use small electrodes with small diameter wires of 20 μm [164] and an electrode array that is considered one of the longest is only 31.5 mm [165]. Another example of a miniaturized and wireless device is an endovascular, wireless, and battery-free millimetric implant that can stimulate specific peripheral nerves that are difficult to reach surgically [166].

Despite these advancements, challenges remain in the field of neuroprosthetics. These include ensuring the long-term stability and biocompatibility of implanted devices, optimizing the specificity and resolution of neural interfaces, and developing more intuitive control strategies for users. In addition, translating neuroprosthetic technologies from research settings to clinical practice requires rigorous testing, regulatory approval, and consideration of ethical and social implications.

Looking ahead, the field of neuroprosthetics holds immense promise for improving the lives of individuals with neurological disorders or injuries. Ongoing research aims to further enhance the functionality and usability of neuroprosthetic devices, incorporating advancements in materials science, machine learning, and neuroscience. As these technologies continue to evolve, they have the potential to revolutionize the way we approach neurological rehabilitation and restoration of function.

## Advancements in Neuroimaging: Transforming Neurosurgery, Neuro-Oncology and Stroke Care

### Overview

As we look forward to breakthroughs in neuroprosthetics, advancements in neuroimaging are equally revolutionizing the field of neurological diagnostics and treatments. The advent of new imaging techniques like CT, nuclear magnetic resonance, PET, and ultrasonic scanning has revolutionized our understanding of the nervous system in both its healthy state and when affected by the disease (Figure 4). These advancements have provided unprecedented clarity and detail in imaging, greatly enhancing our diagnostic capabilities.

### Neuro-Oncology and Neurosurgery

Innovations in neuroimaging have been pivotal in enhancing patient care, significantly reducing morbidity and mortality rates among patients receiving neurosurgical care [167]. The improvement in brain MRI for anatomical mapping has led to rapid growth and progress. This enhancement is important for diagnosing and treating oncological diseases of the nervous system, which include a variety of tumors such as meningiomas. Among the most prevalent primary brain tumors in adults are cerebral gliomas, with an incidence rate of 5 to 6 per 100,000 person-years [168]. At the point of initial diagnosis, the challenge of distinguishing brain tumors from benign lesions is challenging due to their similar appearances on MRI scans. Contrast-enhanced MRI, favored for its superior soft-tissue resolution and accessibility, serves as the primary method for such differentiation. Typically, brain tumor diagnoses rely on conventional MRI techniques, including T1-weighted and T2-weighted sequences. However, these standard imaging approaches sometimes struggle to distinguish between tumor changes due to disease progression and nonspecific, treatment-related alterations, especially after therapeutic interventions. PET scanning, using various radioactive tracers to target distinct metabolic and molecular processes, offers more data that enhance specificity, especially in clinically ambiguous cases. Radiolabeled amino acids in PET scanning become essential in neurodiagnostics, with the Response Assessment in Neuro-Oncology working group recommending its use alongside MRI for comprehensive brain tumor management. Meanwhile, advanced MRI techniques like perfusion-weighted imaging, diffusion-weighted imaging (DWI), and proton magnetic resonance spectroscopic imaging [87] continue to be evaluated clinically for their potential to provide critical physiological or biochemical insights beyond standard MRI capabilities. The evolution of modern neurosurgery and radiology demonstrates the impact of radiological advancements on neurosurgical practices. The pace of these developments is so rapid that newer neurosurgical residents might be unfamiliar with older procedures like pneumoencephalography or the challenges of distinguishing between neurosurgical pathologies before the advent of CT. Moreover, such progress has supported the execution of high-quality clinical trials, improving evidence-based neurosurgical practice.

### Stroke Medicine

Stroke is one of the leading causes of death in older ages and time to treatment is crucial. Over the recent decades, ischemic stroke medicine has evolved with new technological innovations, specifically with the advent of AI and the few critical examples mentioned subsequently are just the tip of the iceberg. Multiple AI-based models are able to detect and segment core infarct, detect LVO, calculate ASPECTS score, and more [169]. One example is a study from 2011, in which a computer-automated detection (CAD) scheme using a circular adaptive region of interest method was developed and implemented on noncontract head CT scans to identify subtle changes in attenuation indicative of ischemic stroke [170]. The findings from the study indicated that CAD significantly enhanced the detection of strokes for emergency physicians and radiology residents [170]. In another study, researchers demonstrated the efficacy of an artificial neural network in distinguishing acute strokes from stroke mimics within 4.5 hours of symptom onset, with a mean sensitivity of 80% and specificity of 86.2% [171]. In the domain of automatic lesion segmentation, a recent study used an ensemble of 2 CNNs to effectively segment DWI lesions, irrespective of their size, while simultaneously mitigating false positives, achieving a Dice score of 0.61 for small lesions and 0.83 for large lesions [172]. In detecting LVOs, a support vector machine (SVM) algorithm demonstrated high sensitivity (97.5%) in identifying the Middle cerebral artery dot sign on noncontrast CT scans in patients with an acute stroke [173]. A commercial software, based on CNN, achieved an accuracy of 86% in detecting proximal LVO, with a sensitivity of 90.1% and specificity of 82.5% [174]. In ASPECTS grading, a commercial software platform offering automated ASPECTS scoring demonstrated comparable performance to neuroradiologists in scoring ASPECTS on noncontrast CT scans in patients with acute stroke (*P*<.003) [175]. In stroke prognosis, a study found that a generalized linear model combining DWI and perfusion-weighted imaging MR outperformed individual modalities in predicting tissue outcomes [176], and another study showed that a CNN trained on MRP source images achieved an area under the curve of 0.871 in predicting final infarct volume [177].

### Neurodegenerative Diseases

The significance of biomarkers in comprehending and diagnosing neurodegenerative diseases is growing. The use of imaging biomarkers for the live examination of these disorders has seen a significant rise in recent decades, offering enhanced diagnostic capabilities and deeper insights into disease mechanisms.

Neurodegenerative diseases, notably AD, are understood to commence years before the manifestation of symptoms. Research, particularly on familial AD, outlines a sequence of pathological events beginning with the buildup of amyloid beta (Aβ), detectable via PET imaging and cerebrospinal fluid analysis, culminating in cognitive deficits and dementia. These processes not only occur in a specific sequence but also overlap over time, offering insights into the disease’s progression. The National Institute on Aging and Alzheimer’s Association has established a research framework for AD diagnosis, using the AT(N) scale to categorize Aβ, tau, and neurodegeneration markers. These markers, identifiable through imaging biomarkers in vivo, enhance the sensitivity and specificity of AD diagnostics, underscoring the vital role of advanced imaging techniques in the early detection and understanding of neurodegenerative diseases. Neuroimaging has become integral to diagnosing suspected neurodegenerative diseases, using various MRI techniques, and developing novel PET ligands. These tools provide objective measures for detecting and monitoring disease presence and progression, aiding in patient care, and facilitating clinical trial recruitment and treatment efficacy evaluation. The cross-disciplinary approach, incorporating imaging biomarkers, is crucial for diagnosing and comprehending neurodegenerative disorders, emphasizing the expanding utility of neuroimaging in medical research and patient management [178]. Aβ PET imaging has transformed the diagnostic landscape for AD, allowing for the in vivo quantification of Aβ plaques, a key AD biomarker. The development of Aβ-specific PET tracers, such as Pittsburgh compound B and subsequent F-18-labeled tracers, has facilitated this advancement [179]. Similarly, tau-specific PET tracers have opened new avenues for diagnosis, prognosis, and clinical trial outcomes in AD, correlating tau pathology with cognitive symptoms and deterioration [180]. The portfolio of PET ligands for identifying biomarkers of neurodegeneration has grown considerably, with some advancing to clinical use and others offering new insights into these conditions. MRI techniques continue to aid diagnosis and enhance our understanding of neurodegenerative diseases, with structural MRI being the most accessible imaging tool. Fluorodeoxyglucose PET, despite its limitations, remains a valuable tool for investigating neuronal injury in dementia [181], illustrating the complex nature of neuroimaging in understanding and treating neurodegenerative diseases.

Few studies also use machine learning to aid diagnosis. For example, an innovative CAD system was developed to diagnose AD from MRI images, based on aging brains and machine learning, and to identify the AD-related regions [182]. The experiments demonstrated that the proposed method could predict AD patients with a competitive accuracy of 92.36%, comparable to existing methods. In another study, the researchers trained a deep learning algorithm based on CNN to predict a diagnosis of AD or mild cognitive impairment, based on fluorodeoxyglucose-PET imaging [183]. The algorithm achieved an impressive area under the receiver operating characteristic curve of 0.98 when predicting the final clinical diagnosis of AD in an independent test set. It demonstrated 82% specificity at 100% sensitivity, with predictions made an average of 75.8 months before the final diagnosis. Notably, the algorithm’s performance surpassed that of human readers, with a sensitivity of 57% and specificity of 91%. Saliency map analysis revealed an attention to known areas of interest, highlighting the entire brain’s importance in the diagnostic process [183]. Another example is PD, a common neurodegenerative disease where early detection is highly important to improve patient’s quality of life [184]. In recent years, remarkable progress has been made in using advanced computational methods in neuroimaging, providing a valuable tool set for the medical imaging research community to extract pertinent features. These methodologies have been instrumental in developing sophisticated diagnostic approaches for PD [184]. In one study neural activity and functional connectivity within the olfactory brain network were investigated [181]. Through the application of independent component analysis and the generalized linear model, discernible differences between patients with PD and healthy controls were identified, with independent component analysis demonstrating superior performance compared with generalized linear model. Similarly, a predictive model using fMRI datasets for PD diagnosis through multiclass patient classification was devised [182]. Feature reduction and selection were achieved using principal-component analysis and the Fisher discriminant ratio, while the classification task was carried out using the least square SVM. Notably, these classifiers exhibited impressive accuracy levels of up to 87.89% and a precision of 82.54%. In another study, resting-state fMRI datasets were used, leveraging an SVM classifier to effectively distinguish 19 patients with PD from 18 healthy controls [183]. In a study aimed at building a model based on Grey’s cerebellum changes, an SVM classifier used data of cerebellar structural changes derived from voxel-based morphometry for PD detection with an accuracy of more than 95% [184]. Researchers also reported that they were able to detect and differentiate successfully patients with PD from healthy ones, by associating different facial expressions and brain activity on fMRI.

In conclusion, the field of medical imaging has witnessed remarkable progress, evolving from the inception of x-ray imaging to the advent of fMRI and other cutting-edge technologies. This trajectory of innovation, coupled with the emergence of AI technologies, has paved the way for groundbreaking applications in the realm of neurological disorders, significantly enhancing our understanding of various neurological conditions.

## Summary

The integration of AI with new medical technological advancements has ushered in a transformative era for neurology, reshaping diagnostic, therapeutic, and research landscapes. With the advent of EHR systems and the widespread adoption of telemedicine, neurologists now have unparalleled access to patient data and the ability to deliver care remotely. This shift streamlines clinical workflows and enhances patient care by enabling precise and timely interventions. As most neurological conditions are chronic and require monitoring, this advancement allows for scaling in treatment. Furthermore, the burgeoning field of predictive modeling, powered by vast databases of EHRs, leverages AI to forecast clinical outcomes, offering personalized treatment strategies.

The paradigm shift from traditional physical examinations to reliance on technological data has significantly impacted patient triage and clinical management. Innovations, such as advanced imaging technologies, revolutionized neurological diagnostics, providing deep insights into the brain’s anatomy and function. Stroke prevalence led to the dire need for rehabilitation in neurology, while new wearable devices and robotics in rehabilitation have further expanded the horizons of patient care, offering targeted therapy that adapts to the dynamic needs of individuals and saves expensive hospital visits. These technological advancements highlight the evolving approach to neurology, emphasizing the importance of integrating cutting-edge tools for improved diagnosis, treatment, and patient outcomes. AI models that analyze brain imaging to detect strokes, coupled with predictive models for conditions like ALS and AD, demonstrate the potential of machine learning to improve patient care and bring to fruition personalized medicine in neurology. The collaboration between neurology and AI technologies paves the way for breakthroughs in understanding and treating neurological disorders, marking a significant leap toward advancing neuroscientific research.

Limitations are present across all these transformative ideas, particularly in advanced imaging technologies such as fMRI, PET, and CT, where challenges include cost, accessibility, and resolution constraints. Another example of the limitations is AI algorithms and their potential biases or hallucinations, data requirements, and the challenges in integrating those into clinical practice or ensuring patient privacy. In genomics, the issues are ethical concerns, off-target effects, the high cost of these technologies, and more.

In summary, the intersection of neurology with emerging technologies has fundamentally changed the landscape of neurological practice. From enhancing diagnostic accuracy and streamlining patient care to personalizing treatment strategies, the entire neurology field stands at the forefront of this revolution. The integration of AI, advanced imaging, and telemedicine underscores its dynamic evolution, driven by the pursuit of excellence in patient care and neuroscientific discovery.

## Abbreviations

AD: Alzheimer disease
ADNI: Alzheimer’s Disease Neuroimaging Initiative
AI: artificial intelligence
ALS: amyotrophic lateral sclerosis
ASPECTS: Alberta Stroke Program Early CT Scores
Aβ: amyloid beta
BCI: brain-computer interface
CAD: computer-automated detection
CNN: convolutional neural network
CNS: central nervous system
CRISPR: Clustered Regularly Interspaced Short Palindromic Repeats
CT: computed tomography
DBS: deep brain stimulation
DWI: diffusion-weighted imaging
EEG: electroencephalogram
EHR: electronic health record
fMRI: functional magnetic resonance imaging
GWAS: genome-wide association study
IMNM: immune-mediated necrotizing myopathy
LVO: large vessel occlusion
MRI: magnetic resonance imaging
MS: multiple sclerosis
NGS: next-generation sequencing
NIH: National Institutes of Health
PD: Parkinson disease
PET: positron emission tomography
scRNA-seq: single-cell RNA sequencing
SeLECT: severity of the stroke, large artery atherosclerosis, early seizure, cortical involvement, and territory of the middle cerebral artery
SVM: support vector machine
